# Supplementary material for: Fate-mapping and functional dissection reveal perilous influence of type I interferon signaling in mouse brain aging
Source: Mol Neurodegener. 2024 Jun 18;19:48. doi: 10.1186/s13024-024-00736-6 (PMC11184889; doi:10.1186/s13024-024-00736-6)
Supplement: Supplementary file 2 — Supplementary Material 2 [file 13024_2024_736_MOESM2_ESM.docx]

**Supplemental Figures and Legends**

**Fate-mapping and functional dissection reveal perilous influence of type I interferon signaling in mouse brain aging**

Ethan R. Roy^1^*, Sanming Li^1^, Sepideh Saroukhani^2,3^, Yanyu Wang^1^, Wei Cao^1^*

^1^Department of Anesthesiology, Critical Care and Pain Medicine, McGovern Medical School, University of Texas Health Science Center at Houston, Houston, TX, USA.

^2^Division of Clinical and Translational Sciences, Department of Internal Medicine, McGovern Medical School, The University of Texas Health Science Center at Houston, Houston, TX, USA.

^3^Biostatistics/Epidemiology/Research Design, Center for Clinical and Translational Sciences, The University of Texas Health Science Center at Houston, Houston, TX, USA.

*Correspondence:

[Wei.Cao@uth.tmc.edu](mailto:Wei.Cao@uth.tmc.edu); [Ethan.R.Roy@uth.tmc.edu](mailto:Ethan.R.Roy@uth.tmc.edu)

6431 Fannin St., University of Texas Health Science Center at Houston, Houston, TX, 77030

**
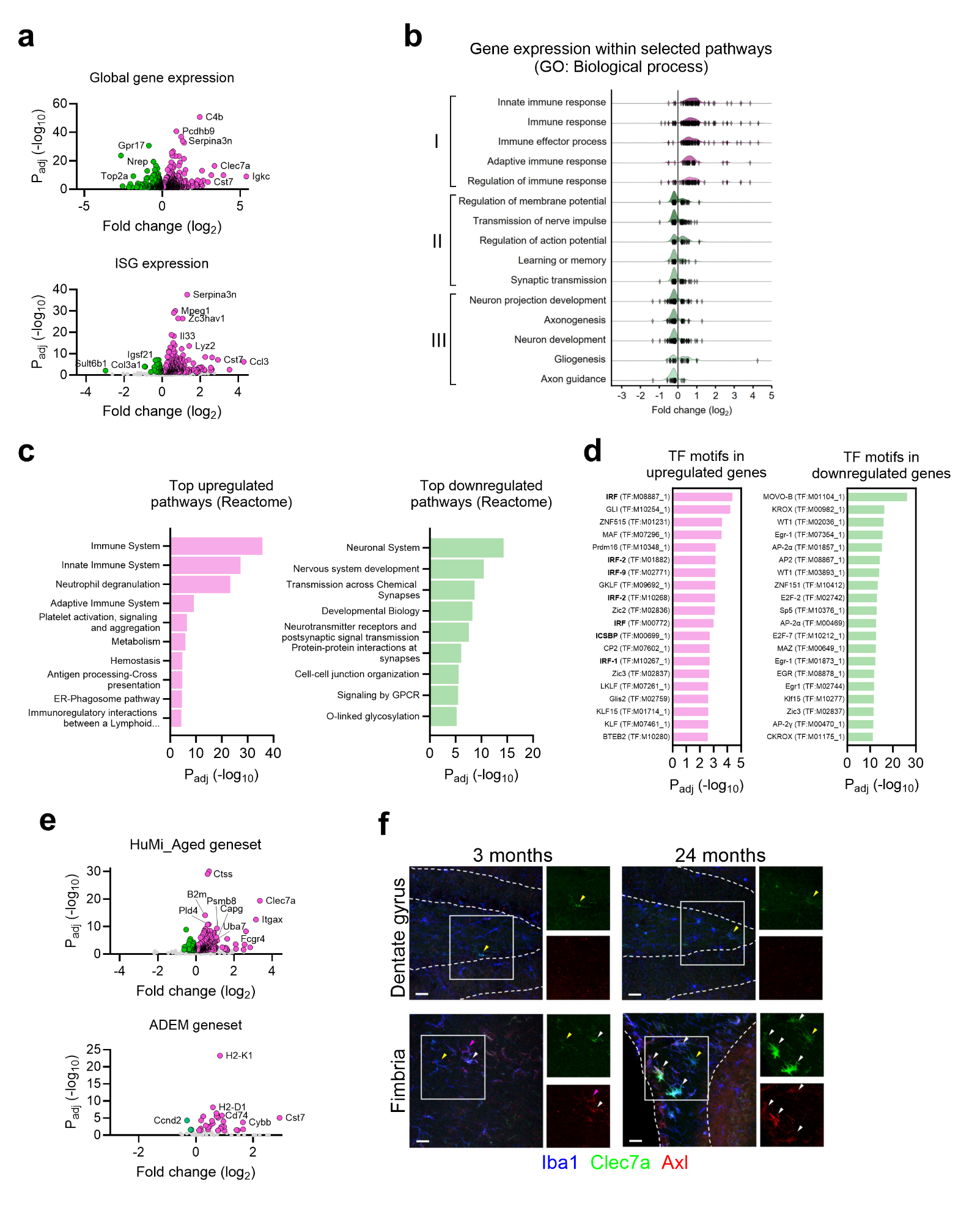
Figure S1**

**Figure S1**

**a**, Volcano plots showing global gene expression changes (*top*) and of ISGs (*bottom*) in bulk cortical tissue of aged animals (24 months, *n* = 8) compared to young (3 months, *n* = 10).

**b**, Ridgeline plots of selected pathways (GO:BP) from each node circled in Fig. 1a. Each plot shows the fold change of DEGs belonging to the pathway (black diamonds) and a colored histogram showing density of DEGs along the axis. Pink histograms indicate pathways with mostly upregulated DEGs and green histograms contain mostly downregulated DEGs.

**c**, Top pathways (Reactome, sorted by significance) enriched in up- (*left*) and downregulated (*right*) DEGs (*P_adj_*<0.05) in aged brains (24 months, *n* = 8) compared to young (3 months, *n* = 10).

**d**, Top 20 TF motifs enriched in up- (*left*) and downregulated (*right*) DEGs (*P_adj_*<0.05) in aged brains (24 months, *n* = 8) compared to young (3 months, *n* = 10). Terms related to IRF are bolded.

**e**, Volcano plots showing expression changes in the HuMi_aged geneset (*top*) and in ADEM genes (*bottom*) in bulk cortical tissue of aged animals (24 months, *n* = 8) compared to young (3 months, *n* = 10).

**f**, Representative images of microglial reactivity markers in dentate gyrus (*top*) and fimbria (*bottom*) of young (3 months, *n* = 8) and aged (24 months, *n* = 8) mice revealing multiple subtypes of reactive microglia, such as Iba1^+^Clec7a^+^ (yellow arrowheads), Iba1^+^Axl^+^ (magenta arrowheads), and Iba1^+^Clec7a^+^Axl^+^ cells (white arrowheads). Isolated Clec7a and Axl channels of the same magnification at right. Scale bars, 25 µm.

**
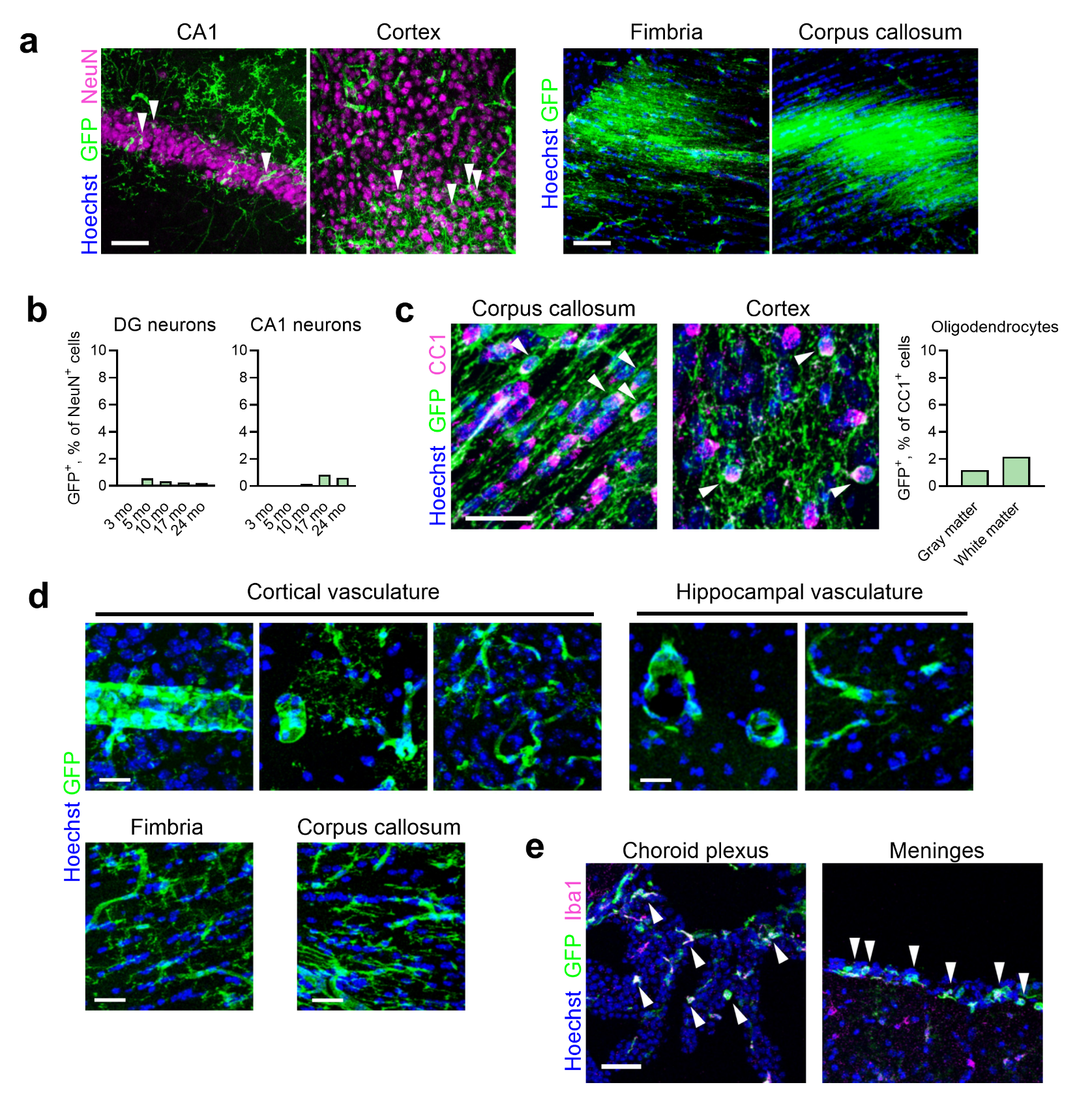
Figure S2**

**Figure S2**

**a**, Further examination of Mx1-GFP^+^ neurons. Representative images (24 months, *n* = 12 animals) of NeuN^+^ neurons in CA1 and superficial cortical layers (*left*; arrowheads), and of white matter axon bundles in fimbria and corpus callosum (*right*). Scale bars, 50 µm.

**b**, Quantification of GFP^+^ percentages of all neurons by age and hippocampal region (DG, *n* = 9,746 cells across ages; CA1, *n* = 4,908 cells across ages. 3 months, *n* = 4 animals; 5 months, *n* = 4 animals; 10 months, *n* = 2 animals; 17 months, *n* = 4 animals; 24 months, *n* = 12 animals).

**c**, Further examination of Mx1-GFP^+^ oligodendrocytes. Representative images (24 months, *n* = 12 animals) of CC1^+^ oligodendrocytes in white matter (corpus callosum; arrowheads) and gray matter (cortex; arrowheads). Scale bars, 25 µm. Quantification of GFP^+^CC1^+^ cells as a percentage of total CC1^+^ cells in gray and white matter of 24-month-old animals (gray matter, *n* = 505 cells; white matter, *n* = 507 cells).

**d**, Further examination of Mx1-GFP^+^ vasculature. Representative images (24 months, *n* = 12 animals) of blood vessels of varying diameters in gray matter regions (*top*; cortex and hippocampus) and white matter regions (*bottom*). Scale bars, 25 µm.

**e**, Examination of Mx1-GFP^+^ border-associated macrophages (BAMs). Representative images (24 months, *n* = 12 animals) of Iba1^+^ BAMs in choroid plexus (*left*; arrowheads) and meninges (*right*; arrowheads). Scale bars, 25 µm.

**
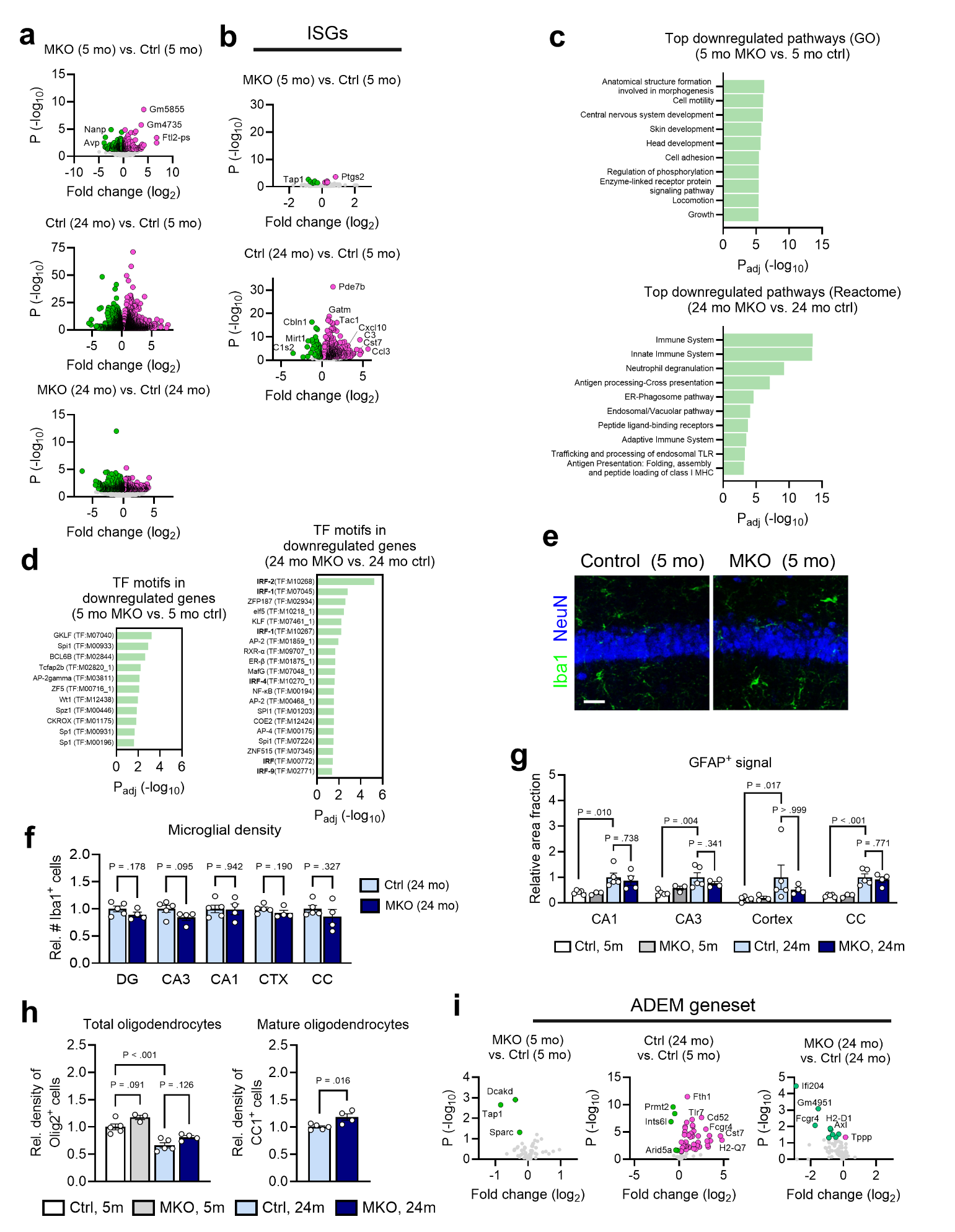
Figure S3**

**Figure S3**

**a**, Volcano plots of global gene expression changes in aged control versus young control brains (*above left*), young MKO versus young control brains (*above right*), and in aged MKO versus aged control brains (*below*).

**b**, Volcano plots of ISG expression changes in young MKO versus young control brains (*above*) and aged control versus young control brains (*bottom*).

**c**, Top pathways (Reactome, sorted by significance) enriched in downregulated DEGs (*P*<0.05) in aged MKO brains (24 months, *n* = 5) compared to aged controls (24 months, *n* = 6).

**d**, Top 20 TF motifs enriched in downregulated DEGs (*P*<0.05) in aged MKO brains (24 months, *n* = 5) compared to aged controls (24 months, *n* = 6). Terms related to IRF are bolded.

**e**, Representative images of Iba1^+^ microglial reactivity in young control (5 months, *n* = 5) and young MKO (5 months, *n* = 3) brains (CA1 region shown). Scale bar, 25 µm.

**f**, Quantification of microglial cell density in multiple regions for aged control (*n* = 5) and aged MKO (*n* = 4) brains.

**g**, Quantification of GFAP^+^ reactive astrocyte area fraction, relative to aged control animals, across various brain regions. Ctrl (5 months), *n* = 5; MKO (5 months), *n* = 3; Ctrl (24 months), *n* = 5 animals; MKO (24 months), *n* = 4 animals. Data represent means and s.e.m.

**h**, Quantification of Olig2 and CC1, markers of total and mature oligodendrocytes, respectively, expressed as relative cell density in the corpus callosum. Ctrl (5 months), *n* = 5; MKO (5 months), *n* = 3; Ctrl (24 months), *n* = 5 animals; MKO (24 months), *n* = 4 animals. Data represent means and s.e.m.

**i**, Volcano plots of ADEM gene expression in aged control versus young control brains (*above left*), aged MKO versus aged control brains (*above right*), and young MKO versus young control brains (*below*).

**
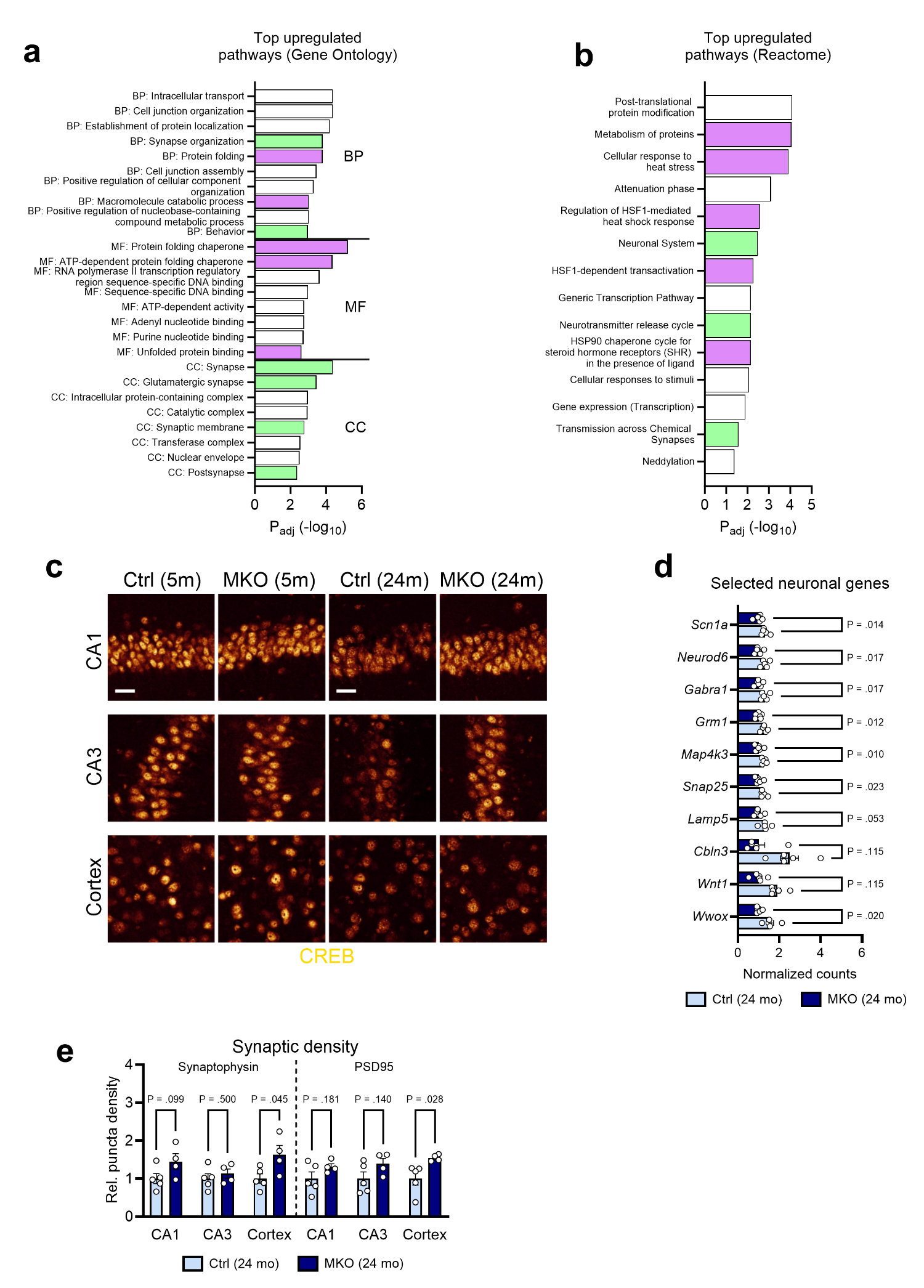
Figure S4**

**Figure S4**

**a**, Top pathways (GO classes, sorted by significance) enriched in upregulated DEGs (*P*<0.05) in aged MKO brains (24 months, *n* = 5) compared to aged controls (24 months, *n* = 6). BP, Biological Process; MF, Molecular Function; CC, Cellular Compartment. Green bars indicate pathways relating to neuronal function, and purple bars indicate pathways relating to protein folding.

**b**, Top pathways (Reactome, sorted by significance) enriched in upregulated DEGs (*P*<0.05) in aged MKO brains (24 months, *n* = 5) compared to aged controls (24 months, *n* = 6). Green bars indicate pathways relating to neuronal function, and purple bars indicate pathways relating to protein folding.

**c**, Representative images of CREB in CA1, CA3 and cortex of young and aged control MKO brains. Scale bars, 25 µm. Ctrl (5 months), *n* = 5 animals; MKO (5 months), *n* = 3 animals; Ctrl (24 months), *n* = 5 animals; MKO (24 months), *n* = 4 animals.

**d**, Expression data for genes related to neuronal function between MKO (24 months, *n* = 5) and aged controls (24 months, *n* = 6). Data represent means of normalized counts and s.e.m.

**e**, Quantification of synaptic puncta densities, including synaptophysin^+^ pre-synapses and PSD95^+^ post-synapses, in various regions of control (24 months, *n* = 5 animals) and MKO (24 months, *n* = 4 animals) brains. Data represent means of normalized counts and s.e.m.

**
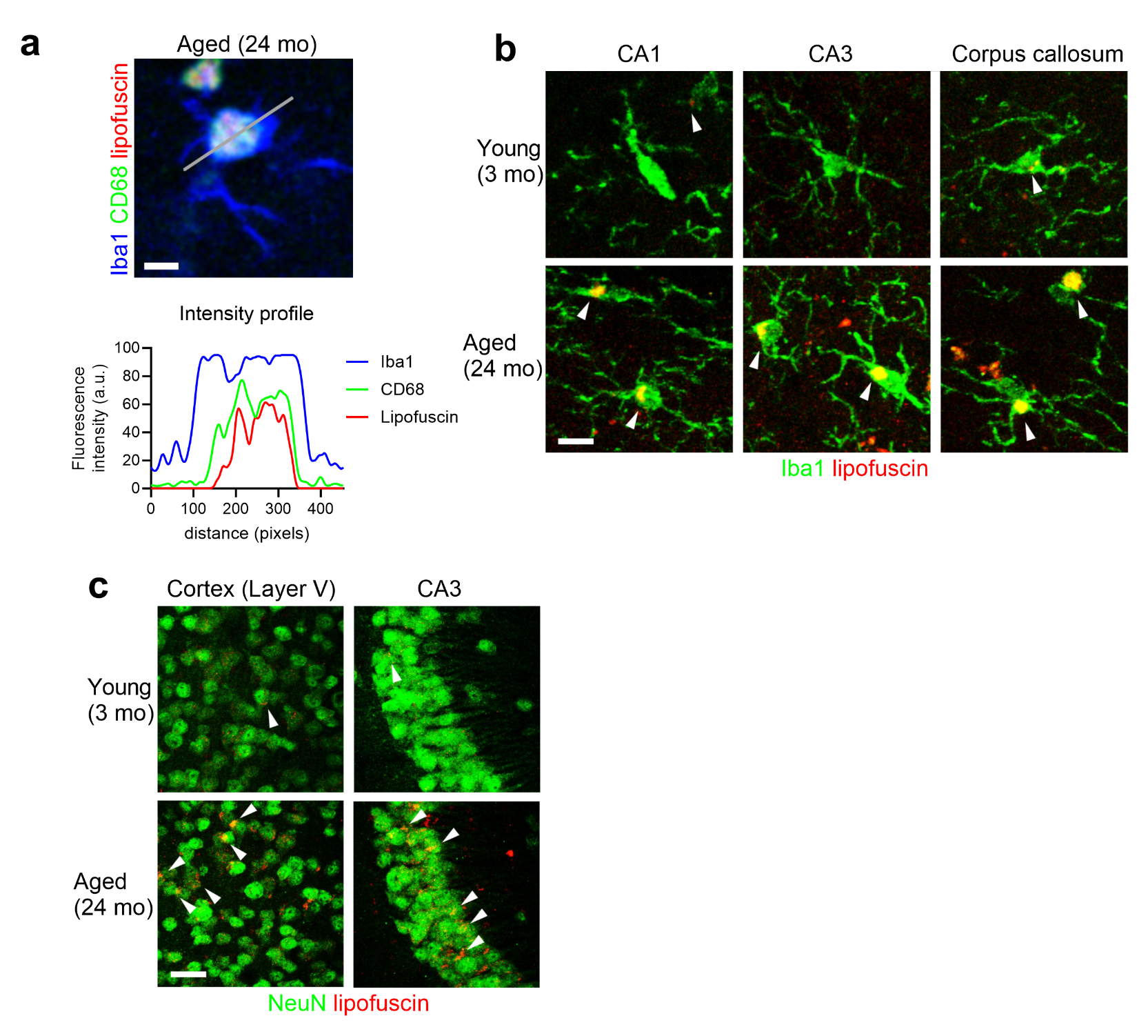
Figure S5**

**Figure S5**

**a**, Representative high-magnification image (*top*) of a microglia in aged brain containing lipofuscin within CD68^+^ lysosomal compartment. Scale bar, 5 µm. Intensity profile (*bottom*) of three channels from the gray line on the image (*top*), showing the red lipofuscin signal completely contained within the green CD68 signal.

**b**, Representative images of microglial lipofuscin accumulation (arrowheads) in various regions of young (3 months, *n* = 8) and aged (24 months, *n* = 8) brains. Scale bar, 10 µm.

**c**, Representative images of neuronal lipofuscin accumulation (arrowheads) in various regions of young (3 months, *n* = 8) and aged (24 months, *n* = 8) brains. Scale bar, 20 µm.
